# Metabolomic and lipidomic signatures of *Helicobacter pylori* infection: Unveiling biochemical pathways associated with neurodegeneration

**DOI:** 10.1002/imo2.70088

**Published:** 2026-02-25

**Authors:** Meenakshi Kandpal, Tarun Prakash Verma, Siddharth Singh, Hamendra Parmar, Hem Chandra Jha

**Affiliations:** ^1^ Mehta family School of Biosciences and Biomedical Engineering Indian Institute of Technology Indore Indore Madhya Pradesh India; ^2^ School of Biotechnology Devi Ahilya University Indore Madhya Pradesh India

## Abstract

Our study employed an integrated lipidomics and metabolomics approach to elucidate *Helicobacter pylori*‐driven metabolic perturbations along the gut–brain axis. *H. pylori* infection was established in gastric epithelial (AGS) cells, and the resulting conditioned media (secretome) was collected and exposed to neuronal (IMR‐32) cells. Gastric, neuronal, and secretome samples were then harvested for the extraction of metabolites and lipids. Comprehensive molecular profiling was performed using high‐resolution LC–MS/HRMS. The acquired data were analysed through lipidomics and metabolomics pipelines to identify significantly altered metabolites and lipid species. Subsequent statistical and pathway enrichment analyses uncovered dysregulated metabolic pathways. Finally, network‐based interaction analysis integrated disease‐associated metabolites to identify key molecular hubs potentially linking *H. pylori* infection to neuronal metabolic dysfunction and neurodegenerative processes.

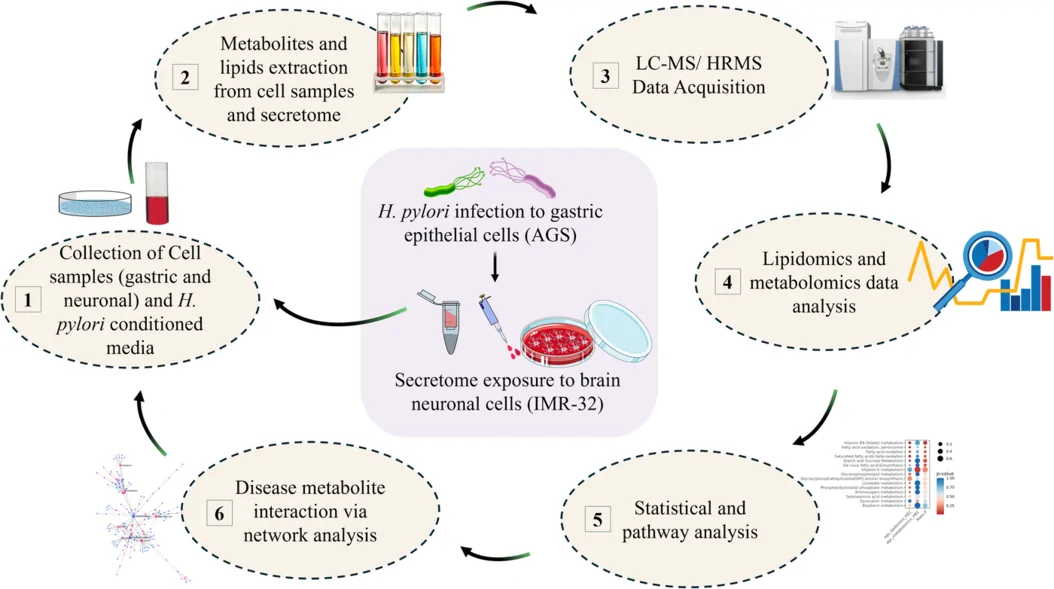

‘Omics’ technologies have transformed our understanding of disease mechanisms and hold significant promise for discovering diagnostic biomarkers and early detection, particularly in neuroinfectious and neurodegenerative disorders [1]. Neurometabolomics, the comprehensive profiling of low‐abundance metabolites, is particularly suited to reveal subtle perturbations in neuronal energy, redox, and neurotransmitter pathways that underpin central nervous system (CNS) dysfunction. Because the CNS depends on tightly regulated bioenergetic homoeostasis, even modest shifts in metabolic flux can propagate into synaptic failure, neuroinflammation, and progressive neurodegeneration, highlighting the need for integrated metabolomic and lipidomic approaches to map these changes at a systems level [2]. Evidence suggests that metabolic dysregulation plays a crucial role in the pathophysiology of several neurological disorders [3]. *Helicobacter pylori* (*H. pylori*) is a Gram‐negative bacterium that infects nearly half of the world's population, with the majority of cases, about 80% remaining asymptomatic [4]. Nevertheless, around 20% of those infected develop clinical manifestations such as gastritis, duodenitis, or peptic ulcer disease, and approximately 2% further progress to gastric cancer [5]. In addition to its well‐established role in gastrointestinal diseases, *H. pylori* infection has been implicated in the development of systemic disorders, potentially contributing to neurological abnormalities by promoting chronic inflammation throughout the body. Epidemiological studies report a higher prevalence of *H. pylori* seropositivity and the presence of pathogen‐specific antibodies in cerebrospinal fluid of patients with Alzheimer's disease (AD), suggesting systemic dissemination and possible CNS involvement [6]. Wang et al. demonstrated that the intraperitoneal administration of *H. pylori* culture filtrates preincubated with N2a cells elicited robust AD‐like pathology in Sprague‐Dawley rats, characterised by tau hyperphosphorylation at Thr205, Thr231, and Ser404, concomitant with pathological activation of the tau‐regulatory kinase GSK‐3β [7]. Additionally, our group has demonstrated that *H. pylori* infection and its secretome are associated with AD through the activation of the JAK/STAT signalling pathway [8]. Collectively, these findings suggest that chronic *H. pylori* infection and its secreted factors can induce AD‐like neuronal alterations by activating GSK‐3β and JAK/STAT signalling, key mediators of tau pathology and neuroinflammation.

Within the multi‐omics landscape, lipidomics is particularly relevant because neuronal membranes are highly enriched in complex phospholipids, sphingolipids, and cholesterol, which orchestrate synaptic vesicle trafficking, receptor clustering, and signal transduction [9]. Perturbations in phosphoinositides, gangliosides, and DHA‐derived neuroprotective lipids are tightly linked to oxidative stress, neuroinflammation, and neurodegeneration, making a combined metabolomic lipidomic readout well‐suited to capture gut‐brain axis disturbances driven by chronic *H. pylori* infection and its antimicrobial resistance‐associated secretome. The clinical challenge posed by *H. pylori* is exacerbated by rising antimicrobial resistance, which compromises eradication regimens and promotes persistent infection [10]. Resistant isolates exhibit distinct metabolic adaptations that enhance survival under antibiotic pressure and may qualitatively alter the composition and biological activity of the bacterial secretome [11]. However, the role of antimicrobial resistance status in shaping the metabolic crosstalk between *H. pylori*, gastric epithelium, and the nervous system remains poorly defined. To address this gap, the present study investigates how clinical *H. pylori* isolates with divergent antibiotic susceptibility profiles, triple‐resistant, semi‐resistant, and triple‐sensitive, remodel the metabolomic and lipidomic landscape of human gastric epithelial (AGS) cells and neuronal (IMR‑32) cells exposed to their conditioned media. The experimental workflow of the present study is shown in Figure S1.

By integrating untargeted metabolomics and lipidomics with pathway‐level meta‐analysis, this work delineates strain‐specific reprogramming of various pathways. This experimental gut–brain axis model enables dissection of how antibiotic resistance‐associated secretomes differentially perturb neuronal energy metabolism, antioxidant defences, and membrane signalling networks, providing mechanistic insight into the potential contribution of drug‐resistant *H. pylori* infection to neurodegenerative and neuropsychiatric disease risk.

## 1 MOLECULAR SIGNATURES IN NEURONAL CELLS EXPOSED TO *H. PYLORI* SECRETOME

To elucidate strain‐specific molecular alterations induced by *H. pylori* (HB10, HJ9, HB1), we conducted untargeted metabolomic and lipidomic profiling of AGS cells post‐infection (MOI 100) using LC–MS. Data analysis *via* MetaboAnalyst revealed distinct dysregulation patterns, with significant strain‐dependent shifts in metabolites and lipid species. Comprehensive metabolomic profiling of gastric epithelial cells exposed to both antibiotic‐resistant (*H. pylori* strains HB10 and HB1) and antibiotic‐sensitive (HJ9) strains revealed a mix of shared and unique metabolic changes (Figure S2, Tables S1–S12).

Exposure of IMR‐32 cells to secretome from distinct *H. pylori* strains induced unique, strain‐specific metabolic alterations. Cells exposed to HB1 conditioned media (CM) representing the secretome, exhibited significant upregulation of metabolites, including glutathione (log_2_FC = 4.99; FDR = 0.07), indicating substantial oxidative burden in HB1 CM exposed neurons; ubiquinol‐8 (log_2_FC = 0.73; FDR = 0.01), a critical electron transport chain component; Cer(d18:1/12:0) (log_2_FC = 0.63; FDR = 0.02), a stress‐responsive sphingolipid, pointed to heightened mitochondrial respiratory demands and ceramide‐mediated apoptotic signalling. Additionally, we observed significant downregulation of N‐Acetyl‐b‐glucosaminylamine (log_2_FC = −0.68; FDR = 0.01) (Figure 1A, Table S13). HB10 CM exposure led to increased levels of 5‐hydroxyconiferyl alcohol (log_2_FC = 0.08; FDR = 0.02), phosphocreatine (log_2_FC = 5.12; FDR = 0.05, retinyl ester (log_2_FC = 0.29; FDR = 0.02) significantly. The significant reduction in deoxyadenosine abundance (log₂FC = −3.01; FDR = 0.0102) is indicative of dysregulated purine nucleotide metabolism, which may compromise retinoid‐linked signalling pathways and limit DNA replication and repair processes (Figure 1B, Table S14).

**Figure 1:**
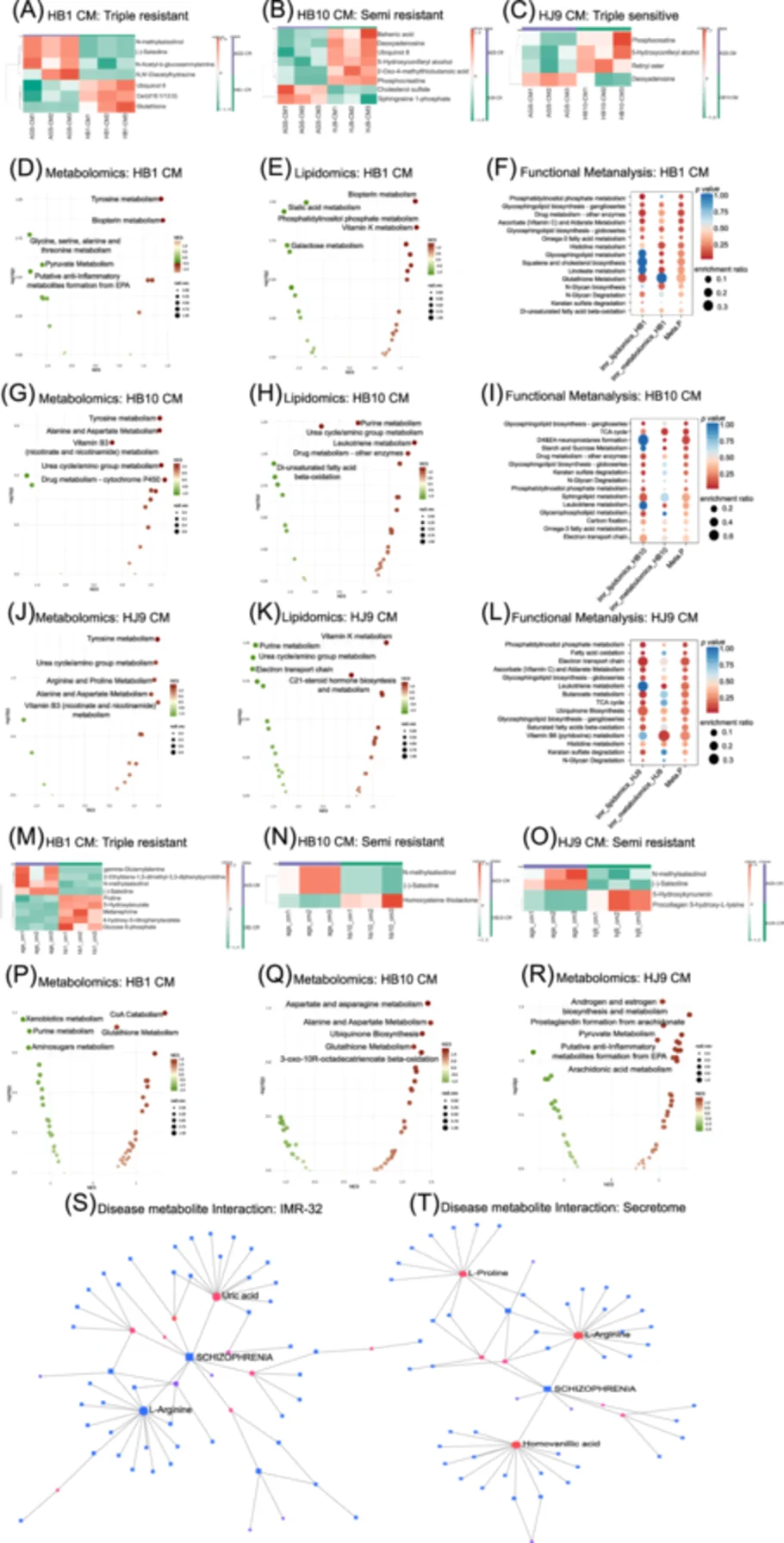
Metabolites altered in neuronal cells exposed to the secretome of resistant and sensitive *H. pylori* strains. (A–C) Heatmaps showing the top significantly altered metabolites in IMR‐32 cells exposed for 24 h to conditioned media derived from AGS cells infected with HB1 (triple‐resistant), HB10 (semi‐resistant), or HJ9 (triple‐sensitive) compared with the AGS‐CM control. Red indicates relative upregulation and green downregulation of metabolites across biological replicates, highlighting distinct secretome‐specific metabolic signatures, including glutathione and ubiquinol‑8 enrichment in HB1‐CM, retinyl ester and deoxyadenosine changes in HB10‐CM, and phosphocreatine elevation in HJ9‐CM. (D–L) Bubble plots of pathway enrichment from neuronal metabolomics (left) and lipidomics (middle), with integrated functional meta‐analysis (right) for HB1 CM (D–F), HB10 CM (G–I), and HJ9 CM (J–L) exposures. The *x*‑axis denotes normalised enrichment score (NES), and the *y*‑axis denotes pathway category; bubble size reflects the number of hits per pathway, and colour encodes adjusted *p*‑value (FDR). HB1 CM primarily perturbs phosphatidylinositol phosphate metabolism, glycosphingolipid biosynthesis, biopterin and ascorbate metabolism, whereas HB10 CM additionally disrupts the TCA cycle, urea cycle/amino group metabolism, and leukotriene pathways; HJ9‐CM shows more localised effects on tyrosine metabolism, vitamin B3 metabolism, and urea cycle pathways. Heatmaps of disease‐relevant neuronal metabolites significantly altered by HB1 CM (M), HB10 CM (N), and HJ9 CM (O) compared with control. Consistent depletion of salsoline and N‑methylsalsolinol is observed across all three secretome conditions, while HB1 CM uniquely induces strong glutathione accumulation and HB10 CM and HJ9‐CM differentially modulate deoxyadenosine, phosphocreatine, and other energy‐ and neurotransmission‐related metabolites. Altered metabolic pathways associated with polar metabolites in *H. pylori* secretome for HB1 CM (P), HB10 CM (Q), and HJ9 CM (R). Disease metabolite interaction in the neural milieu (S) and secretome (T). Network visualisation of metabolite‐pathway‐disease associations reveals the systems‐level metabolic dysfunction in *H. pylori*‐exposed neuronal cells and *H. pylori* secretome. Blue squares represent disease nodes, with “SCHIZOPHRENIA” shown as the central blue square that connects multiple regions of the network. Large red circles denote key metabolite hubs such as L‐arginine and uric acid, which show numerous links to the schizophrenia node and to other neighbours, indicating metabolites with high connectivity and potential central roles in the disease context. Medium‐pink circles correspond to additional metabolites associated either directly with schizophrenia or indirectly via metabolite hubs, forming secondary clusters around the major red nodes. Small purple circles mark more peripheral metabolites that have only one or a few connections, reflecting more isolated or weaker associations within the network. The thin grey lines connecting these nodes represent the underlying disease–metabolite or metabolite–metabolite interactions captured in your analysis.

In contrast, cells exposed to HJ9 CM showed elevated levels of unique metabolites, including behenic acid (log_2_FC = 1.34; FDR = 0.02), 2‐oxo‐4‐methylthiobutanoic acid (log_2_FC = 2.78; FDR = 0.09) (Figure 1C, Table S15). Importantly, a robust and statistically significant depletion of salsoline and N‐methylsalsolinol was consistently detected across all secretome‐exposed conditions. The strain‐independent depletion of these metabolites implies either activation of a conserved host‐mediated neuroprotective response or the presence of shared bacterial determinants that perturb salsolinol metabolic pathways, highlighting their potential utility as mechanistically informative biomarkers of neurological dysfunction.

Figure 1D–F shows the functional analysis of metabolites, the functional analysis of lipids, and the functional metanalysis of combined lipidomics and metabolomics in neuronal cells exposed to HB1 CM (resistant). It was observed that Phosphatidylinositol phosphate metabolism (Meta.P = 0.096), Glycosphingolipid biosynthesis – ganglioseries (Meta.P ≈ 0.098), and ascorbate (Vitamin C) and aldarate metabolism were the top‐most altered pathways (Tables S16–S18). Due to the critical roles of phosphoinositides in neurotransmitter release, synaptic plasticity, and phospholipase C‐mediated Ca²⁺ signalling, together with gangliosides' functions in axonal outgrowth and neuronal survival [12], these data implicate HB1 secretome in extensive remodelling of neuronal membrane signalling systems and redox equilibrium. Figure 1G–I illustrates functional analysis of metabolites, functional analysis of lipids, and functional metanalysis of combined lipidomics and metabolomics in neuronal cells exposed to HB10 CM (resistant). We observed that Glycosphingolipid biosynthesis – ganglioseries, TCA cycle (Meta.P = 0.0661), and D4&E4‐neuroprostanes formation (Meta.P = 0.0910) were key altered pathways (Tables S19–S21). These findings are consistent with impaired mitochondrial ATP generation and reduced formation of DHA‐derived neuroprotective eicosanoids, required for synaptic neurotransmitter synthesis and reuptake [13]. These collectively imply a heightened vulnerability of semi‐resistant strain‐exposed neurons to bioenergetic failure and inflammatory damage. Figure 1J–L illustrates the functional analysis of metabolites, lipids, and a combined functional metanalysis of lipidomics and metabolomics in neuronal cells exposed to HJ9 CM (sensitive). It was observed that Phosphatidylinositol phosphate metabolism, fatty acid oxidation, and the electron transport chain were the top‐most altered pathways (Tables S22–S24). These patterns strongly suggest that the triple‐sensitive HJ9 strain elicits localised, reversible metabolic adjustments rather than the widespread network collapse observed with resistant strains.

Additionally, we performed metabolomic and lipidomic profiling of *H. pylori* secretome (HB10 CM, HJ9 CM, and HB1 CM) to identify the distinct metabolites secreted in *H. pylori* conditioned media. Numerous metabolites, such as 4‐hydroxy‐3‐nitrophenylacetate (log_2_FC = 2.49; FDR = 0.004), metanephrine (log_2_FC = 4.28; FDR = 0.01), 5‐hydroxyisourate (log_2_FC = 3.68; FDR = 0.0001), proline (log_2_FC = 1.91; FDR = 0.06), were upregulated in HB1 secretome (Figure 1M, Table S25). In HB10 CM, we observed the significant downregulation of salsoline and N‐methylsalsolinol expression (log_2_FC = −0.96976; FDR = 0.011509) (Figure 1N, Table S26). However, HJ9 secretome showed the upregulation of procollagen 5‐hydroxy‐l‐lysine (log_2_FC = 3.73; FDR = 0.09) (Figure 1O, Table S27). Consistent with the alterations detected in secretome‐exposed neuronal cells, we observed a uniform, significant downregulation of salsoline and N‐methylsalsolinol expression. Figure 1P illustrates the functional analysis of metabolites in the HB1 secretome. It was observed that CoA Catabolism, Glutathione Metabolism, and Purine Metabolism were the top‐most altered pathways (Table S28). Figure 1Q shows the functional analysis of metabolites in HB10 (resistant) secretome. Aspartate and asparagine metabolism, Alanine and Aspartate Metabolism, and Ubiquinone Biosynthesis were the top‐most altered pathways (Table S29). Figure 1R illustrates the functional analysis of metabolites in the HJ9 (sensitive) secretome. It was observed that Androgen and oestrogen biosynthesis and metabolism, Prostaglandin formation from arachidonate, and pyruvate metabolism were the top‐most altered pathways (Table S30). Disease metabolite interaction network analysis in neuronal cells exposed to *H. pylori* secretome revealed strain‐specific metabolic perturbations associated with distinct pathological conditions. However, in neuronal cells, exposure to the *H. pylori* secretome prominently elevated L‐arginine and uric acid, both of which are tightly associated with schizophrenia‐related networks, indicating a redox imbalance and nitric oxide dysregulation. Also, it induced broader disruptions in neurotransmitter‐linked pathways and oxidative stress mediators (Figure 1S and 1T).

Our metabolomic profiling revealed strain‐specific metabolic perturbations in gastric and neuronal cells (Figure S3). Resistant strains (HB10, HB1) elicited the most extensive neuronal metabolic remodelling, characterised by overwhelming oxidative stress (glutathione accumulation), disrupted membrane signalling (phosphoinositide and ganglioside dysregulation), and mitochondrial energy crisis, whereas HJ9 primarily altered tyrosine, folate, vitamin K, and β‐alanine metabolism. In neuronal cells, resistant strains activated PGE₂–STAT1/STAT3 inflammatory signalling, while sensitive strains enhanced oxidative dopamine metabolism, both decreased neuroprotective dopamine derivatives. Network mapping revealed disturbances in the BH4/biopterin, salsolinol, and vitamin K‐sphingolipid pathways, suggesting impaired neurotransmitter synthesis, oxidative stress, and loss of neuroprotective signalling. These alterations overlapped with metabolic hubs associated with schizophrenia and neuroinflammation, indicating convergence on key lipid and monoamine pathways.

Moving forward, we will validate and quantitatively profile the key metabolites identified in gastric and neural compartments using murine models of *H. pylori* infection. High‐resolution lipidomic and metabolomic analyses will define infection‐induced metabolic perturbations in both gastric tissue and the CNS. This integrated *in vivo* framework is expected to clarify how *H. pylori*‐driven metabolic reprogramming and lipid dysregulation contribute to neurodegenerative phenotypes.

## AUTHOR CONTRIBUTIONS

**Meenakshi Kandpal** and **Hem Chandra Jha:** Conceptualisation. **Meenakshi Kandpal** and **Tarun Prakash Verma:** Data analysis; data interpretation. **Meenakshi Kandpal, Tarun Prakash Verma, Siddharth Singh,** and **Hem Chandra Jha:** Formal analysis; visualisation. **Meenakshi Kandpal:** Resources; writing—original draft. preparation. **Meenakshi Kandpal, Tarun Prakash Verma, Hamendra Parmar,** and **Hem Chandra Jha:** Writing—review & editing. **Hem Chandra Jha:** Supervision; validation; data analysis; data interpretation. All authors have read the final manuscript and approved it for publication.

## CONFLICT OF INTEREST STATEMENT

The authors declare the following potential conflict of interest: a patent application (**IITIIR&D/Patent/2 11/2025‐26/01)** has been filed related to the data and findings presented in this manuscript. This patent is held by the **Indian Institute of Technology Indore** by **Hem Chandra Jha** and **Meenakshi Kandpal**, who may benefit commercially from the results described herein. All authors have disclosed this information and have no other conflicts of interest.

## AI STATEMENT

The manuscript was only checked for grammar and language using Grammarly, and no AI tools were used for writing, analysis, or content creation.

## ETHICS STATEMENT

No animals or humans were involved in this study.

## Supporting information

The online version contains supplementary methods, figures, and tables available.
**Figure S1:** Experimental workflow for omics‐based analysis of *H. pylori*‐induced metabolic and neurological alterations.
**Figure S2:** Metabolomic and lipidomic profiling of *H. pylori*‐infected gastric epithelial cells reveals differential metabolic dysregulation stratified by antibiotic susceptibility status.
**Figure S3:** Distinct metabolic changes in the gastric and neural compartment upon exposure of resistant and sensitive *H. pylori* strains and their respective secretome.

**Table S1:** Identified metabolites (polar and non‐polar) in gastric epithelial cells infected with HB1.
**Table S2:** Identified metabolites (polar and non‐polar) in gastric epithelial cells infected with HB10.
**Table S3:** Identified metabolites (polar and non‐polar) in gastric epithelial cells infected with HJ9.
**Table S4:** Altered metabolic pathways identified by functional analysis of polar metabolites in gastric epithelial cells infected with HB1.
**Table S5:** Altered metabolic pathways identified by functional analysis of non‐polar metabolites in gastric epithelial cells infected with HB1.
**Table S6:** Altered metabolic pathways identified by functional meta‐analysis of gastric epithelial cells infected with HB1.
**Table S7:** Altered metabolic pathways identified by functional analysis of polar metabolites in gastric epithelial cells infected with HB10.
**Table S8:** Altered metabolic pathways identified by functional analysis of non‐polar metabolites in gastric epithelial cells infected with HB10.
**Table S9:** Altered metabolic pathways identified by functional meta‐analysis of gastric epithelial cells infected with HB10.
**Table S10:** Altered metabolic pathways identified by functional analysis of polar metabolites in gastric epithelial cells infected with HJ9.
**Table S11:** Altered metabolic pathways identified by functional analysis of non‐polar metabolites in gastric epithelial cells infected with HJ9.
**Table S12:** Altered metabolic pathways identified by functional meta‐analysis of gastric epithelial cells infected with HJ9.
**Table S13:** Identified metabolites (polar and non‐polar) in neuronal cells exposed to HB1 CM.
**Table S14:** Identified metabolites (polar and non‐polar) in neuronal cells exposed to HB10 CM.
**Table S15:** Identified metabolites (polar and non‐polar) in neuronal cells exposed to HJ9 CM.
**Table S16:** Altered metabolic pathways identified by functional analysis of polar metabolites in neuronal cells exposed to HB1 CM.
**Table S17:** Altered metabolic pathways identified by functional analysis of non‐polar metabolites in neuronal cells exposed to HB1 CM.
**Table S18:** Altered metabolic pathways identified by functional meta‐analysis of neuronal cells exposed to HB1 CM.
**Table S19:** Altered metabolic pathways identified by functional analysis of polar metabolites in neuronal cells exposed to HB10 CM.
**Table S20:** Altered metabolic pathways identified by functional analysis of non‐polar metabolites in neuronal cells exposed to HB10 CM.
**Table S21:** Altered metabolic pathways identified by functional meta‐analysis of neuronal cells exposed to HB10 CM.
**Table S22:** Altered metabolic pathways identified by functional analysis of polar metabolites in neuronal cells exposed to HJ9 CM.
**Table S23:** Altered metabolic pathways identified by functional analysis of non‐polar metabolites in neuronal cells exposed to HJ9 CM.
**Table S24:** Altered metabolic pathways identified by functional analysis of non‐polar metabolites in neuronal cells exposed to HJ9 CM.
**Table S25:** Identified metabolites (polar and non‐polar) in *H. pylori* secretome of HB1 CM.
**Table S26:** Identified metabolites (polar and non‐polar) in *H. pylori* secretome of HB10 CM.
**Table S27:** Identified metabolites (polar and non‐polar) in *H. pylori* secretome of HJ9 CM.
**Table S28:** Altered metabolic pathways identified by functional analysis of polar metabolites of *H. pylori* secretome of HB1 CM.
**Table S29:** Altered metabolic pathways identified by functional analysis of polar metabolites of *H. pylori* secretome of HB10 CM.
**Table S30:** Altered metabolic pathways identified by functional analysis of polar metabolites of *H. pylori* secretome of HJ9 CM.

## Data Availability

Supplementary materials (results, methods, figures, tables, graphical abstract, slides, videos, Chinese translated version, and updated materials) may be found in the online DOI or iMetaOmics http://www.imeta.science/imetaomics/. Raw files of the data have been uploaded to the repository OSF Data Repository https://osf.io/67n4e/.
