# Supplementary material for: Metabolomic and lipidomic signatures of Helicobacter pylori infection: Unveiling biochemical pathways associated with neurodegeneration
Source: IMetaOmics. 2026 Feb 25;3(2):e70088. doi: 10.1002/imo2.70088 (PMC13674429; doi:10.1002/imo2.70088)
Supplement: Supplementary file 1 [file IMO2-3-e70088-s002.docx]

**Supporting Information to Metabolomic and Lipidomic Signatures of *Helicobacter pylori* Infection: Unveiling Biochemical Pathways associated with Neurodegeneration**

**Running title**: Metabolic Signatures of *Helicobacter pylori-*mediated Neurodegeneration

Meenakshi Kandpal^1^**,** Tarun Prakash Verma^1^, Siddharth Singh^1^, Hamendra Parmar^2^ and Hem Chandra Jha^1*^

^1^Mehta family School of Biosciences and Biomedical Engineering, Indian Institute of Technology Indore, Khandwa Road, Simrol, Indore, M.P., 453552, India

^2^School of Biotechnology, Devi Ahilya University, Indore, M.P., 453552, India

^*^Correspondence: [hemcjha@iiti.ac.in](mailto:hemcjha@iiti.ac.in) (Hem Chandra Jha)

**1. Material and methods**

**1.1. Cell culture**

The AGS cell line, which originates from human gastric adenocarcinoma, and the IMR-32 cell line, derived from human neuroblastoma, were obtained from the National Centre for Cell Science located in Pune, India. These cell lines were cultured in Dulbecco’s modified Eagle’s medium (DMEM), supplied by Himedia Laboratories Pvt. Limited, India, and supplemented with 10% fetal bovine serum (FBS), also obtained from Himedia Laboratories Pvt. Limited, India. The culture medium was further supplemented with 50 U/mL penicillin, 100 μg/mL streptomycin, and 2 mM L-glutamine. The cells were grown in a humidified incubator at 37°C with 5% CO₂ [1].

**1.2. Bacterial Isolates and Culture**

Clinical strains of *Helicobacter pylori* were isolated from gastric biopsy (HB) and gastric juice (HJ) samples obtained from patients showing symptoms of gastritis, following a standard protocol. Individual bacterial colonies were inoculated into 3 mL of brain heart infusion medium (BD Difco brain heart infusion broth; catalogue number 237500), which was supplemented with 10% fetal bovine serum (FBS; Himedia; catalog number RM 10432) and a 1× concentration of antibiotics specific for *H. pylori* (10 mg/l vancomycin, 5 mg/L amphotericin B, 5 mg/L cefsulodin, and 5 mg/L trimethoprim). The cultures were prepared in snap-cap tubes (BD; catalogue number 352001) and incubated in a microaerobic workstation (Whitley DG250) to create microaerophilic conditions (85% N₂, 10% CO₂, and 5% O₂) at 37°C.

**1.3. Antibiotic susceptibility test of *H. pylori* isolates**

The minimum inhibitory concentrations (MIC) of 10 clinical *H. pylori* isolates (HJ1, HJ9, HJ10, HJ17, HJ14, I10, HB10, HB14, HB1, and HB5) towards antibiotics used in concomitant quadruple therapy, such as clarithromycin, metronidazole, and amoxicillin, were assessed using the broth microdilution method as per the protocol mentioned [2]. The following antibiotic concentrations were used to check the susceptibility: Clarithromycin (0, 0.25, 0.5, and 1 ug/mL), metronidazole (0, 4, 8, and 16 ug/mL), and amoxicillin (0, 0.0625, 0.125 and 0.25 ug/mL). Resistance was defined in accordance with the European Committee on Antimicrobial Susceptibility Testing (EUCAST) guidelines: clarithromycin (MIC > 0.5 μg/mL), metronidazole (MIC > 4 μg/mL), and amoxicillin (MIC > 0.125 μg/mL). We used a complete brain-heart infusion (BHI) medium as a control. Based on susceptibility profiles, three strains were selected: HB1 (triple-resistant to all three antibiotics), HB10 (semi-resistant), and HJ9 (triple-sensitive).

**1.4. Infection methodology**

To begin the infection process, 2.2 x 10^6^ AGS cells were plated in 100 mm cell culture dishes. When the cells reached 80–85% confluency, they were infected with *H. pylori* strains HB10, HJ9, and HB1 at a multiplicity of infection (MOI) of 100. The MOI was calculated using a method described in a previous study. The bacterial concentration (CFU/mL) was estimated based on the final optical density (OD), and the volume of bacterial culture needed for infection was determined accordingly. Prior to each infection, the identity of the bacterial strains was verified through duplicate gram staining, following a standardized procedure.

Following the infection protocol, gastric epithelial cells were exposed to three different clinical isolates of *H. pylori*: HB10, HJ9, and HB1. Uninfected AGS cells served as the control group. After incubating for 24 hours, the conditioned media from the infected cells were collected and referred to as *H. pylori*-derived conditioned media (HPCM) in the study. Neuronal cells (IMR-32) were then treated with a mixture of cDMEM and HPCM in a 7:3 ratio for 24 hours **(Fig S1).**

**1.5. Sample Preparation and LC-MS Analysis**

AGS cell lysates, IMR-32 cell lysates, and collected secretome (conditioned media) from each condition were processed for metabolomics and lipidomics analysis. Cell samples were rapidly quenched by addition of ice-cold methanol. Metabolite and lipid fractionation was achieved using a methanol:chloroform:water mixture (1:1:0.9, v/v/v) following established liquid-liquid extraction protocols. The aqueous upper phase (polar metabolites) and organic lower phase (lipids) were transferred to separate tubes. Organic fractions were concentrated using a rotational vacuum concentrator (RVC 2-18 CDplus; Martin Christ, Germany), and aqueous fractions were further dried by freeze-drying (Alpha 1-2 LDplus; Martin Christ, Germany). Conditioned media samples were lyophilized prior to polar metabolite extraction using the same solvent system.

**1.5.1.** **Lipidomics Analysis:** Lipid-containing organic fractions were redissolved in chloroform:methanol (1:1, v/v) and analyzed using a Dionex Ultimate 3000 HPLC system coupled to a Bruker MicrOTOF QII high-resolution mass spectrometer (HRMS) [3]. Chromatographic separation was performed using a C₁₈ reverse-phase column (XBridge 5 μm C₁₈, 4.6 × 250 mm; Waters, USA). Mobile phase A was deionized distilled water:methanol (60:40, v/v) with 0.1% formic acid; mobile phase B was methanol:isopropanol (1:9, v/v) with 0.1% formic acid. The LC gradient employed was: 0% B at 0 min, 20% B at 1 min, 100% B at 16–20 min, and 0% B at 20.1–31 min, with a flow rate of 0.4 mL/min. Quality control and blank samples were prepared. MS data were acquired in centroid mode between m/z 80–700 and m/z 500–3000 in ESI positive mode with reference mass correction enabled.

**1.5.2. Metabolomics Analysis:** Samples were redissolved in water:methanol (1:1, v/v). Mobile phase A consisted of 0.1% formic acid in distilled water, and mobile phase B was 0.1% formic acid in acetonitrile. The LC gradient program was: 95% B at 0 min, 95% B at 0.5 min, 40% B at 10.5–15 min, and 95% B at 17–42 min, with a flow rate of 0.25 mL/min. MS data were acquired in centroid mode between m/z 80–700 in electrospray ionization (ESI) positive mode. Pooled quality control (QC) samples were prepared from equal volumes of all experimental samples; blank samples contained solvent mixture only.

## **1.6. Data Processing and Statistical Analysis**

Raw LC-MS data were converted to mzXML format using Bruker Compass Data Analysis 5.1 (Bruker Daltonics, USA). All analyses were performed in R using MetaboAnalystR for statistical analysis, and functional analysis with OptiLCMS used for LC-MS raw spectral processing [4].

**1.6.1. LC-MS raw data processing and feature table construction**

LC-MS files were further processed using an ROI-based workflow tuned on pooled QC injections. QC files were used for ROI extraction with stringent retention time indexing and contaminant removal enabled. Peak picking and alignment parameters were optimized using the HPLC-Q/TOF platform setting, and the optimized parameters were applied to all samples for peak profiling after raw data import. Features were annotated in positive ion mode with an absolute m/z tolerance of 0.015, then formatted without isotopic and adduct filtering and with permissive missingness retention to preserve candidate signals for downstream filtering. Annotation tables, aligned peak tables, and peak summaries were exported for subsequent analyses. Data integrity checks were applied, followed by minimum value replacement for missing entries, then prevalence filtering retaining features meeting a 25 percent presence criterion. Quantile normalization was used for the one factor statistical workflow.

Prior to statistical analysis, ANOVA assumptions were rigorously validated through: (1) visual assessment of Q-Q plots to assess normality of data distribution, (2) boxplot inspection to evaluate homogeneity of variances across groups, and (3) confirmation of independent observations across the study design.

Comparative statistical analysis of metabolites between groups employed one-way ANOVA with Benjamini-Hochberg false discovery rate (FDR) correction (q < 0.05) to account for multiple hypothesis testing. Tukey's honest significant difference (HSD) post hoc test was used for pairwise comparisons to identify specific differences between groups. Non-parametric Kruskal-Wallis testing was performed as a confirmatory analysis for metabolites with potential violations of ANOVA assumptions, providing robust statistical validation independent of distribution assumptions. Heatmaps of top significantly altered metabolites (both polar and non-polar) were generated to visualize metabolic profiles and treatment-specific clustering patterns.

Global structure and group separation were assessed using a row scaled heatmap with Euclidean distance and Ward clustering. Multivariate modelling was performed using PLS-DA with score plots, loading plots, and VIP based variable importance visualisations, alongside biplots. Predictive performance was evaluated using a five-fold PLSDA cross-validation routine with accuracy as the performance metric and classification plots exported.

**1.6.2. Functional analysis from peaks and cross-omics pathway meta-analysis**

Functional analysis was performed using a peaks to pathways strategy implemented via the mummichog and GSEA framework, enabling pathway activity prediction directly from LC-MS features without requiring complete metabolite identification. A formatted mass table was analyzed with instrument parameters set to positive ion mode, adduct consideration enabled, and m/z tolerance set to 0.02, with retention time included in seconds. After preprocessing and quantile normalization, pathway enrichment used a GSEA based peak enrichment method and was run against the human pathway library hsa_mfn, then peaks to pathways plots were exported. To integrate metabolomics and lipidomics at the pathway level, Functional Meta-analysis was conducted by importing both datasets, applying median normalization with log transformation and autoscaling, and mapping both datasets under consistent positive mode settings. Datasets were jointly included and combined using a Fisher based meta-analysis configured as fixed effect with stringent significance thresholding, and pathway bubble plots were generated with explicit p-value and overlap cutoffs and a capped number of displayed pathways.

Comparisons included: (i) AGS gastric epithelial cells infected with HB1 (triple-resistant), HB10 (semi-resistant), or HJ9 (triple-sensitive) *H. pylori* strains versus uninfected AGS control; (ii) IMR-32 neuronal cells exposed to HB1 CM, HB10 CM, or HJ9 CM versus AGS-CM control; and (iii) *H. pylori* secretome (HB1 CM, HB10 CM, HJ9 CM versus AGS CM control.

Pathway enrichment analysis was performed to identify biologically relevant metabolic pathways altered by *H. pylori* infection and/or bacterial secretome exposure. Enrichment analysis was conducted separately for gastric epithelial cells (AGS) and neuronal cells (IMR-32) to capture compartment-specific metabolic responses. Pathways with q (FDR) < 0.1 were considered statistically significant and prioritized for biological interpretation.

To comprehensively examine the interplay between polar metabolites and lipid mediators, integrated functional meta-analysis was performed to combine metabolomics and lipidomics datasets at the pathway level. Differentially abundant metabolites and lipids identified from univariate analysis were aggregated using Gene Set Enrichment Analysis (GSEA, version 1) with Fisher's method for p-value integration. The Homo sapiens Metabolic Functional Network (MFN) pathway library was employed as the reference pathway database. This integrated approach allowed simultaneous evaluation of metabolic and lipid signalling dysregulation across both compartments. Statistical significance for integrated analysis was set at q (FDR) < 0.1 to permit discovery of pathway-level effects that may be individually modest but collectively meaningful.

Metabolite-disease interaction networks were constructed for three distinct biological contexts: (i) the gastric epithelial compartment (AGS cells), (ii) the neuronal compartment (IMR-32 cells), and (iii) the bacterial secretome (conditioned media). Network visualization mapped functional relationships between identified metabolites/lipids, dysregulated biological pathways, and known disease-associated processes. This network-based approach enabled identification of metabolic hub molecules and pathway interconnections that may mechanistically link gastric epithelial dysfunction to neuronal alterations through bacterial-derived secretory factors, thereby elucidating the molecular basis of the gut-brain axis perturbations associated with antibiotic-resistant *H. pylori* infection.

**DATA AVAILABILITY STATEMENT**

The Raw data files have been uploaded to the OSF Data Repository at <https://osf.io/67n4e/>.

**2. Results: Molecular Signatures associated with *H. pylori* infection in gastric epithelial cells**

AGS cells infected with distinct *H. pylori* strains induced unique, strain-specific metabolic alterations. Cells infected with HB1, exhibited significant upregulation of metabolites, including LysoPC (18:1/0:0) (log_2_FC = 7.9198; FDR = 0.013566), Pantothenic acid (log_2_FC = 3.1729; FDR = 0.022655) **(Figure S2A, Table S4).** HB10 infection led to increased levels of Clupanodonyl carnitine (log_2_FC = 3.2285; FDR = 0.00012354), PE(P-16:0/16:0) (log_2_FC = 1.6763; FDR = 0.0027849) significantly **(Figure S2B, Table S5).** In contrast, HJ9 infection showed elevated levels of unique metabolites, including 4-Guanidinobutanamide (log_2_FC = 2.3879; FDR = 0.083321), 13'-hydroxy-alpha-tocotrienol (log_2_FC = 1.8713; FDR = 0.086661) **(Figure S2C, Table S6).** Importantly, a robust and statistically significant depletion of anserine was consistently detected across antibiotic-resistant bacterial-infected conditions (HB1 and HB10). Also, significant depletion of Codeine-6-glucuronide was consistently detected across all infected conditions. **Figure S2D** shows the functional analysis of metabolites, the functional analysis of lipids, and the functional metanalysis of combined lipidomics and metabolomics in gastric epithelial cells infected with HB1 (resistant). It was observed that Vitamin B9 (folate) metabolism, Fatty acid oxidation, and Starch and Sucrose Metabolism were the top-most altered pathways. **Figure S2E** illustrates functional analysis of metabolites, functional analysis of lipids, and functional metanalysis of combined lipidomics and metabolomics in gastric epithelial cells infected with HB10 (resistant). We observed that Urea cycle/amino group metabolism, Arachidonic acid metabolism, and Glycerophospholipid metabolism were the top-most altered pathways. **Figure 2SF** illustrates the functional analysis of metabolites, lipids, and a combined functional metanalysis of lipidomics and metabolomics in gastric epithelial cells infected with HJ9 (sensitive). It was observed that Linoleate metabolism, Vitamin B9 (folate) metabolism, and Glycosylphosphatidylinositol (GPI)-anchor biosynthesis were the top-most altered pathways.

Disease metabolite interaction network analysis in AGS cells infected with *H. pylori* strains HB1, HB10, and HJ9 revealed strain-specific metabolic perturbations associated with distinct pathological conditions **(Figure S2G).** HB1 induced prominent neuroactive shifts with elevated interactions involving glycine, linking to schizophrenia and Alzheimer’s disease. HB10 predominantly affected steroid and amino acid metabolism, with metabolites like testosterone and ornithine connected to endocrine and mitochondrial dysfunctions. HJ9 exhibited intermediate alterations, reflecting both neuropsychiatric and metabolic disease associations. These findings suggest that *H. pylori* modulates host metabolic profiles in a strain-dependent manner, potentially influencing extra-gastric disease susceptibility through metabolite–disease axis perturbation.

**Supplementary Figures**

**Figure S1. Experimental workflow for omics-based analysis of *H. pylori*-induced metabolic and neurological alterations.** AGS gastric epithelial cells were seeded and infected with three clinical strains of *Helicobacter pylori* (HB1: triple-resistant, HB10: semi-resistant, HJ9: triple-sensitive) at a multiplicity of infection (MOI) of 100 for 24 hours (Step 1). Parallel collection of both infected cell pellets and *H. pylori*-derived conditioned media (HPCM) was performed. Cell pellets underwent lipid and metabolite extraction using methanol:chloroform:water (1:1:0.9, v/v/v) for subsequent metabolomics and lipidomics analysis. Conditioned media (Step 2) was collected and sterile-filtered for transfer to neuronal cells. IMR-32 neuronal cells were exposed to HPCM at a 7:3 ratio (culture medium:HPCM) for 24 hours (Step 3) to model the gut-brain axis interaction. Cell pellets from neuronal cultures underwent identical lipid and metabolite extraction. Both cell-derived and conditioned media samples were analyzed using liquid chromatography-mass spectrometry (LC-MS) coupled to a Dionex Ultimate 3000 HPLC and Bruker MicrOTOF QII high-resolution mass spectrometer to simultaneously quantify polar and non-polar metabolites and lipid mediators across the gastric epithelial and neuronal compartments.

**Figure S2. Metabolomic and lipidomic profiling of *H. pylori*-infected gastric epithelial cells reveals differential metabolic dysregulation stratified by antibiotic susceptibility status**

**(A–C)** Heatmaps of altered metabolites in AGS cells infected with *H. pylori* strains: Hierarchical clustering heatmaps depict the top significantly altered polar and non-polar metabolites in AGS gastric epithelial cells following 24-hour infection with HB1 (triple-resistant, panel A), HB10 (semi-resistant, panel B), and HJ9 (triple-sensitive, panel C) strains. Red coloration indicates upregulated metabolites, while green indicates downregulated metabolites. Each row represents a unique metabolite.

**(D–F) Metabolomic and lipidomic pathway enrichment analysis:** Pathway charts illustrate the functional consequences of metabolic dysregulation. **(D)** HB1-infected AGS cells exhibit marked dysregulation of vitamin metabolism (vitamin B5, D3, K, B9), sphingolipid pathways, and carnitine shuttle metabolism (metabolomics, left panel). Lipidomic analysis reveals enrichment in fatty acid metabolism and carnitine-mediated pathways (middle panel). Integrated pathway analysis identifies vitamin metabolism and lipid signaling as the primary functional modules altered in response to triple-resistant *H. pylori* infection (right panel). **(E)** HB10 infection (semi-resistant phenotype) induces intermediate metabolic responses affecting urea cycle metabolism, arachidonic acid signaling, and glycerophospholipid remodeling. **(F)** HJ9 (triple-sensitive) infection demonstrates restricted metabolic alterations concentrated on folate metabolism, amino acid homeostasis, and inflammatory mediator formation. Bubble size in pathway enrichment plots is proportional to the number of identified metabolites per pathway; bubble color intensity represents the false discovery rate (FDR)-adjusted p-value (darker blue indicates p < 0.05). Pathway enrichment significance threshold: p < 0.05 for univariate analysis, q < 0.1 for integrated meta-analysis **(G)** Metabolite-disease interaction network of AGS cells. Network visualization of metabolite-pathway-disease associations reveals the systems-level metabolic dysfunction in infected gastric epithelium. Nodes represent identified metabolites (gray circles), dysregulated pathways (blue circles), and gastric disease states (red hexagons). Central metabolic hubs including carnitine, amino acids, lipids, and vitamins serve as integration points connecting multiple dysregulated pathways, suggesting that antibiotic-resistant *H. pylori* infection triggers coordinated disruption of energy metabolism, lipid signaling, and antioxidant defense systems in gastric mucosa.

**Fi****gure S3. Distinct metabolic changes in the gastric and neural compartment upon exposure of resistant and sensitive *H. pylori* strains and their respective secretome.** In the gastric compartment, *H. pylori* infection leads to upregulation (shown in red text) and downregulation (shown in green text) of multiple metabolic pathways, contributing to leaky gut formation by disrupting gut barrier integrity, which, in turn, leads to gut dysbiosis. In the neural compartment, exposure to *H. pylori* secretome resulted in selective metabolic remodeling. Specifically, amino acid metabolism (aspartate and asparagine) and vitamin-related pathways (vitamin K metabolism) were predominantly upregulated (red text), suggesting a compensatory cellular response to microbial stress. Conversely, neurotransmitter-associated metabolic pathways, including those linked to dopamine, serotonin, and GABA biosynthesis, were notably downregulated (highlighted in green). This dysregulation is indicative of impaired neurotransmitter homeostasis and may contribute to neuronal dysfunction, neuroinflammation, and ultimately, the progression of neurodegenerative phenotypes.

**References**

[1]	Kandpal, Meenakshi, Budhadev Baral, Nidhi Varshney, Ajay Kumar Jain, Debi Chatterji, Ajay Kumar Meena, Rajan Kumar Pandey, Hem Chandra Jha. 2024. “Gut-brain axis interplay via STAT3 pathway: Implications of *Helicobacter pylori* derived secretome on inflammation and Alzheimer’s disease”. *Virulence. 15:2303853.* https://doi.org/10.1080/21505594.2024.2303853.

[2]	Kouitcheu Mabeku, Laure Brigitte, Bertrand Eyoum Bille, Cromwell Tepap Zemnou, Lionel Danny Tali Nguefack, Hubert Leundji. 2019. "Broad spectrum resistance in Helicobacter pylori isolated from gastric biopsies of patients with dyspepsia in Cameroon and efflux-mediated multiresistance detection in MDR isolates". *BMC Infectious Diseases.* 19(1):880. https://doi.org/[10.1186/s12879-019-4536-8](https://doi.org/10.1186/s12879-019-4536-8)

[3]	Verma, Tarun Prakash, Sonali Adhikari, Siddharth Singh, Chanchal Rani, Rajesh Kumar, Hem Chandra Jha. 2024. “A comprehensive analysis of metabolomics and lipidomics in areca nut mediated oral submucous fibrosis progression through LCMS and Raman spectroscopy". *Journal of Raman Spectroscopy.* 55: 797-808*.* <https://doi.org/10.1002/jrs.6679>

[4] Pang, Zhiqiang, Lei Xu, Charles Viau, Yao Lu, Reza Salavati, Niladri Basu, Jianguo Xia. 2024. "MetaboAnalystR 4.0: a unified LC-MS workflow for global metabolomics". [*Nature Communications*](https://www.nature.com/ncomms). 15: 3675. https://doi.org/10.1038/s41467-024-48009-6.
